# Orthoweb: a software package for evolutionary analysis of gene networks

**DOI:** 10.18699/vjgb-24-95

**Published:** 2024-12

**Authors:** R.A. Ivanov, A.M. Mukhin, F.V. Kazantsev, Z.S. Mustafin, D.A. Afonnikov, Y.G. Matushkin, S.A. Lashin

**Affiliations:** Institute of Cytology and Genetics of the Siberian Branch of the Russian Academy of Sciences, Novosibirsk, Russia; Institute of Cytology and Genetics of the Siberian Branch of the Russian Academy of Sciences, Novosibirsk, Russia Novosibirsk State University, Novosibirsk, Russia; Institute of Cytology and Genetics of the Siberian Branch of the Russian Academy of Sciences, Novosibirsk, Russia Novosibirsk State University, Novosibirsk, Russia; Novosibirsk State University, Novosibirsk, Russia; Institute of Cytology and Genetics of the Siberian Branch of the Russian Academy of Sciences, Novosibirsk, Russia Novosibirsk State University, Novosibirsk, Russia; Institute of Cytology and Genetics of the Siberian Branch of the Russian Academy of Sciences, Novosibirsk, Russia; Institute of Cytology and Genetics of the Siberian Branch of the Russian Academy of Sciences, Novosibirsk, Russia Novosibirsk State University, Novosibirsk, Russia

**Keywords:** gene networks, evolution, phylostratigraphy, генные сети, эволюция, филостратиграфия

## Abstract

This article introduces Orthoweb (https://orthoweb.sysbio.cytogen.ru/), a software package developed for the calculation of evolutionary indices, including phylostratigraphic indices and divergence indices (Ka/Ks) for individual genes as well as for gene networks. The phylostratigraphic age index (PAI) allows the evolutionary stage of a gene’s emergence (and thus indirectly the approximate time of its origin, known as “evolutionary age”) to be assessed based on the analysis of orthologous genes across closely and distantly related taxa. Additionally, Orthoweb supports the calculation of the transcriptome age index (TAI) and the transcriptome divergence index (TDI). These indices are important for understanding the dynamics of gene expression and its impact on the development and adaptation of organisms. Orthoweb also includes optional analytical features, such as the ability to explore Gene Ontology (GO) terms associated with genes, facilitating functional enrichment analyses that link evolutionary origins of genes to biological processes. Furthermore, it offers tools for SNP enrichment analysis, enabling the users to assess the evolutionary significance of genetic variants within specific genomic regions. A key feature of Orthoweb is its ability to integrate these indices with gene network analysis. The software offers advanced visualization tools, such as gene network mapping and graphical representations of phylostratigraphic index distributions of network elements, ensuring intuitive interpretation of complex evolutionary relationships. To further streamline research workflows, Orthoweb includes a database of pre-calculated indices for numerous taxa, accessible via an application programming interface (API). This feature allows the users to retrieve pre-computed phylostratigraphic and divergence data efficiently, significantly reducing computational time and effort.

## Abstract (ru)

В данной статье описывается Orthoweb (https://orthoweb.sysbio.cytogen.ru/) – программный комплекс, разработанный для вычисления эволюционных индексов, включая филостратиграфические индексы и индексы дивергенции (Ka/Ks) как отдельных генов, так и генных сетей. Индекс филостратиграфического возраста (PAI) позволяет оценить эволюционную стадию появления гена (при этом косвенно оценив приблизительное время его возникновения – так называемый эволюционный возраст) на основе анализа ортологичных генов у близкородственных и дальнородственных таксонов. Кроме того, Orthoweb поддерживает расчет индексов возраста транскриптома (TAI) и дивергенции транскриптома (TDI). Эти индексы важны для понимания динамики экспрессии генов и ее последствий для развития и адаптации организмов. Orthoweb содержит также дополнительные аналитические функции, такие как возможность анализа терминов Gene Ontology (GO), что позволяет проводить функциональное обогащение и связывать эволюционное происхождение генов с биологическими процессами. Помимо этого, доступна возможность анализа обогащения по однонуклеотидным полиморфизмам (SNP), который помогает исследовать эволюционное значение генетических вариантов в конкретных геномных регионах. Одной из ключевых особенностей Orthoweb является интеграция перечисленных индексов с анализом генетических сетей. Программный пакет предлагает расширенные средства визуализации, такие как картирование генетических сетей и графическое представление распределения филостратиграфических индексов элементов сетей, что облегчает интуитивную интерпретацию сложных эволюционных связей. Для упрощения рабочих процессов в Orthoweb включена база данных с предварительно рассчитанными индексами для множества таксонов, доступная через API. Эта функция позволяет эффективно получать готовые данные по филостратиграфическим индексам и индексам дивергенции, значительно сокращая время вычислений.

## Introduction

The evolutionary analysis of gene networks allows the study of the origin and development of biological systems in the context of evolution. One of the key aspects of this analysis is the study of gene age indices, which allows us to determine the temporal framework for the emergence and diversification of genes across different phylogenetic lineages. Phylostratigraphy, a methodology based on estimating the evolutionary age of genes, provides an opportunity to identify ancient and recently emerged genes as well as to understand their functional significance in biological processes (Domazet-Lošo, Tautz, 2008; Tautz, Domazet-Lošo, 2011; Šestak et al., 2013; Xie et al., 2017). The aim of phylostratigraphic analysis is to determine the age of a founder gene by assessing the distribution of its homologous genes in the genomes of organisms belonging to different taxonomic groups. The Phylostratigraphic Age Index (PAI) is used in phylostratigraphy to estimate the time of origin of genes and corresponds to the oldest phylostratum that includes homologous sequences of the target gene

The search for genes with homology restricted to specific taxa is particularly interesting from an evolutionary biology perspective, as several studies have shown that novel genes can play an important role in the emergence of new evolutionary traits and may be associated with the appearance of new morphological features in land plants (Bowles et al., 2020) and multicellular animals (Paps, Holland, 2018). It has also been shown that evolutionarily novel genes are involved in organ development cascades, particularly in brain tissue development (An et al., 2023), and that taxon-specific genes are overrepresented in stress response systems and the immune system (Dornburg, Yoder, 2022). Some researchers have also suggested that taxon-specific genes are associated with ecological specialisation in various taxa (Baalsrud et al., 2018).

However, the classical approach to phylostratigraphy faces several limitations due to the increasing volume of genomic data and the insufficient accuracy of the BLASTP algorithm in identifying homologs. These factors, together with high computational complexity, result in phylostratigraphic analyses of whole genome data using BLASTP taking up to several weeks (Buchfink et al., 2021). Consequently, there is a growing need for the development of new software solutions for phylostratigraphic analysis.

Modern software tools such as fagin (Arendsee et al., 2019), GenEra (Barrera-Redondo et al., 2023) and oggmap (Ullrich, Glytnasi, 2023) offer alternative approaches to phylostratigraphic analysis, allowing researchers to overcome some of the limitations of classical methods. The fagin program, written in R, uses a homology search approach based on identifying syntenic regions in the target genome and then searching for homology in both amino acid and nucleotide sequences. The developers of the GenEra software package have introduced several modifications to the classical method of homology detection in phylostratigraphy by replacing the traditional BLASTP search method with the DIAMOND v2 algorithm. This substitution improves the identification of distant homologs by removing restrictions on the number of top sequence matches during alignment. In addition, GenEra’s developers have incorporated features to assess homology detection error and taxonomic representativeness – a metric that considers the presence of gene homologs in at least one representative species at each intermediate taxonomic level between the most distantly related genus and the target species. The oggmap program (Ullrich, Glytnasi, 2023), implemented as a Python package, is designed to generate orthology maps (orthomaps), or, in other words, phylostratigraphic index values for the age of specified ortholog groups, based on the results of tools such as OrthoFinder (Emms, Kelly, 2019) and eggNOG (Huerta-Cepas et al., 2019). Unlike classical phylostratigraphy, this approach does not include a step for ortholog detection using alignment tools. Instead, it relies on precomputed orthology search results in the form of orthomaps, which are then used to estimate gene age. These orthomaps contain information about the ages of genes within each ortholog group.

However, for comprehensive evolutionary analysis, these tools and approaches require knowledge of programming languages. In addition, most of these software solutions rely on alignment algorithms such as BLAST, the runtime of which can significantly slow down the analysis in certain cases. Finally, the existing implementations for calculating phylostratigraphic indices are currently unable to perform a comprehensive and rapid evolutionary analysis of gene network components. In this article, we present Orthoweb – a software package for the evolutionary analysis of gene networks and individual genes – implemented as a web application and available at https://orthoweb.sysbio.cytogen.ru.

## Materials and methods

Orthoweb has been developed in Java using the Spring framework to implement server-side functionality and the Vue.JS and webix frameworks for the client side. A set of cytoscape.js libraries is used for network visualization. MongoDB is used as the database management system (DBMS) to store data from the KEGG database (taxa, list of orthologs, coding sequences, etc.) and intermediate analysis results, which significantly increases the speed of subsequent work with these data.

A database based on the PostgreSQL DBMS is used to store the calculated indices. Access to the data is provided through REST API technology implemented with the FLASK library (flask.palletsprojects.com). This programmatic interface allows data retrieval from various engineering modelling environments (e. g. Matlab, Octave, Statistica) or standard libraries of scripting programming languages (e. g. R, Python).

## Results

Functionality of Orthoweb

**Calculation of evolutionary age indices of single genes.** The primary function of Orthoweb is the estimation of phylostratigraphic age indices (PAI) of genes.

**Fig. 1.**
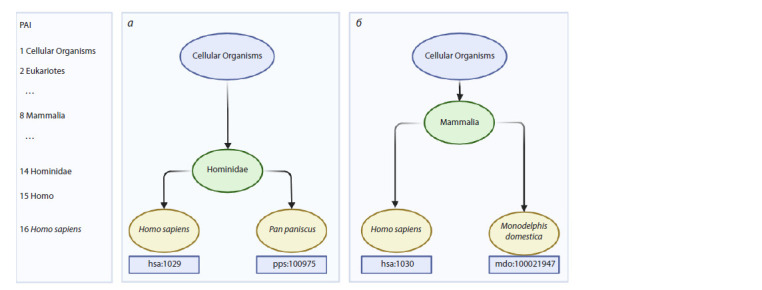
Example of a PAI calculation for two Homo sapiens genes. а – example of an evolutionarily younger gene hsa:1029 (CDKN2A), where the most distantly related organism with an identified ortholog of this gene is Pan paniscus (bonobo chimpanzee); b – example of an evolutionarily older gene hsa:1030 (CDKN2B), where the most distantly related organism with an identified ortholog of this gene is Monodelphis domestica (grey short-tailed opossum). It can be concluded that the gene in example (a) is evolutionarily younger than the gene in example (b). The scale on the left indicates the PAI index, which corresponds to the depth of the taxonomic tree node. Adapted from (Mustafin et al., 2021).

Orthoweb implements two methods to determine PAI: 1) based on the analysis of homology sequence identity metrics and 2) using the classification of proteins into orthologous groups from the KEGG database (KEGG Orthology – KO). Using the KO information from the KEGG database (Kanehisa et al., 2016), Orthoweb allows the identification of orthologs for each protein sequence and determines the species in the genomes of which these orthologs have been found. The taxonomic lineages of the identified species are sequentially compared to the lineage of the studied species to determine their evolutionary ancestry and to determine the most recent common ancestor for a given gene. The position of this ancestor, measured as its distance from the root of the taxonomic tree, is calculated as the PAI (Fig. 1). The taxonomic lineages of orthologs have already been curated in the KEGG database, requiring minimal additional configuration by the user. The calculated PAI indices are stored in a regularly updated database, which is discussed in more detail in the chapter “Database for storing results”. As KEGG orthogroup data is frequently updated, Orthoweb also allows to calculate PAI indices directly from KEGG orthogroups to ensure access to the most up-to-date information. However, such data are not available for all genes. For example, in humans, only about two-thirds of the genes represented in KEGG are associated with KO groups.

The second method for calculating PAI involves using the Best Similarity Table, which is available for the vast majority of genes represented in KEGG (Kanehisa et al., 2016). This method allows users to select homologous genes based on parameters such as the amino acid sequence identity of the proteins encoded by the genes and the results of the Smith– Waterman local sequence alignment algorithm

**Calculation of divergence indices.** Orthoweb also supports the calculation of the ratio of nonsynonymous to synonymous substitutions (the dN /dS ratio) between the sequence of the gene under study and each of its homologs in closely related species, reflected in the Divergence Index (DI). This index allows researchers to determine the type of selection acting on a gene. The index is calculated based on the dN /dS ratio (also referred to as Ka/Ks in the literature), where dN represents the proportion of nonsynonymous substitutions in the sequences of the gene under study and its homologs (i. e. substitutions that result in a change in the amino acid encoded by the codon) and dS represents the proportion of synonymous substitutions (i. e. those that do not result in a change in the encoded amino acid). It is generally accepted that DI values less than 1 indicate that the gene is under purifying selection, values close to 1 suggest neutral evolution, and values greater than 1 imply positive selection (Yang, Nielsen, 2000).

When comparing a single homologous sequence, DI is equivalent to dN /dS. In cases where multiple homologs are present, DI is equal to the average dN /dS value across all comparisons. When calculating the DI index, Orthoweb users can select the taxonomic depth of analysis to account for the evolutionary variability of the gene between organisms with varying evolutionary distances. The calculation of the dN /dS ratio is performed using the PAML software package (Yang, 2007).

**Calculation of gene enrichment with single nucleotide polymorphisms and Gene Ontology term analysis.** Orthoweb also integrates information on Gene Ontology (GO) terms associated with genes and the enrichment of the studied genes with single nucleotide polymorphisms (SNPs). To retrieve information on Gene Ontology terms, Orthoweb uses the resource available at http://geneontology.org/ (Ashburner et al., 2000; Carbon et al., 2021). Data retrieval is performed using the API (application programming interface) provided. For example, a query for the TBP gene is constructed as follows: http://api.geneontology.org/api/bioentity/gene/ NCBIGene:6908/function, specifying the database and the gene identifier within it. Orthoweb provides this information autonomously, relying on associated databases for most model organisms (e. g. TAIR for Arabidopsis thaliana, FlyBase for Drosophila melanogaster, etc.), while for other organisms, it uses the UniProt database. If Gene Ontology contains data for the gene under study and KEGG provides the required identifier – which is true for nearly all well-characterised genes – then identifiers and names of GO terms associated with the gene will be retrieved.

To obtain data on the enrichment of target genes with single nucleotide polymorphisms, an automated query system for the NCBI SNP database (Sayers et al., 2022) is implemented. The query is constructed based on the gene identifier. For example, for the TBP gene with the identifier hsa:6908, the query would take the following form: https://www.ncbi.nlm. nih.gov/snp/?term=6908. As a result of this query, the user will be provided with the number of SNPs found. It should be noted that in the current version of Orthoweb, the SNP search is only implemented for human genes.

**Calculation of evolutionary indices of a group of genes.** Orthoweb also supports the input of gene expression data for the calculation of phylotranscriptomic indices. Phylotranscriptomic index analysis is an approach that integrates information on the evolutionary age of genes with data on their expression levels. This analysis enables the study of the relationship between the PAI index of genes and changes in their activity in the context of different physiological states, adaptive responses or developmental stages of organisms. Using phylotranscriptomic analysis, it is possible to uncover how the evolutionary features of the genome relate to the transcriptional regulation and functional dynamics of genes in different biological contexts. Phylotranscriptomic indices include two evolutionary indices: Transcriptome Age Index (Domazet-Lošo, Tautz, 2010) and Transcriptome Divergence Index (Quint et al., 2012)

The Transcriptome Age Index (TAI) represents the weighted average age of the transcriptome in a given biological process. Expression data serve as an additional multiplier and are used to normalise the result so that the higher the final TAI/TDI value, the greater the contribution of evolutionarily younger/ more variable genes. The formulas used to calculate these indices are as follows:

**Formula. 1.**
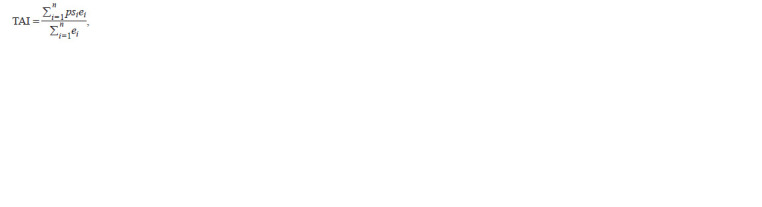
Formula1

where psi is an integer representing the PAI for gene i, ei is the expression level derived from transcriptomic data for gene i, and n is the total number of genes

The Transcriptome Divergence Index (TDI) measures transcriptome divergence and reflects the degree of conservation of a transcriptome in a particular process. This can be used to identify biological processes or development stages in which more conserved, or younger, genes are more highly expressed.

**Formula. 2.**
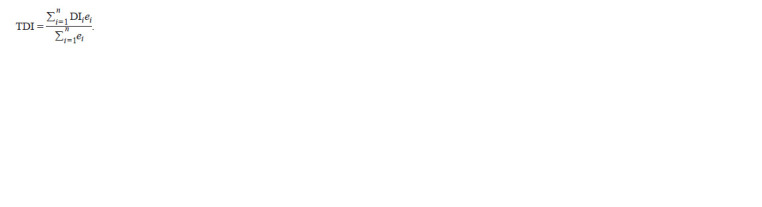
Formula2

where DIi is the divergence index for gene i, ei is the expression level for gene i, n is the total number of genes.

Orthoweb usage examples

To illustrate how Orthoweb works, we will describe its workflow and give examples of its use in phylostratigraphic analysis.

**Fig. 2.**
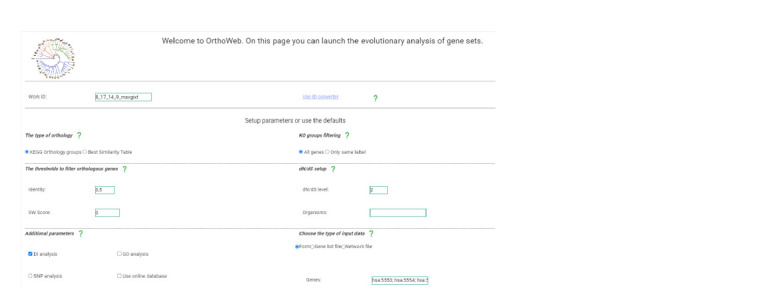
The Start Page of the Orthoweb Web Service.

**Analysis of individual gene characteristics. **When analysing evolutionary indices for single genes, Orthoweb accepts several input file formats: a list of genes entered via a web form, a list of genes uploaded from a file, or a file containing interactions between elements of a gene network in .txt or .tsv format. Users can select the desired input data format in the corresponding form labelled Choose the type of input data (Fig. 2). For accurate analysis in Orthoweb, KEGG gene identifiers must be provided.

The next step involves selecting the analysis mode in the form titled The type of orthology. In this form, you can choose one of two options: calculating phylostratigraphic indices using ortholog family and KO group analysis (the KEGG Orthology groups option) or using homologous sequence analysis (the Best Similarity Table option).

When selecting the KEGG Orthology groups mode, it is also necessary to decide whether to include paralogous genes in the analysis by configuring the KO groups filtering option.

When selecting the mode for calculating phylostratigraphic indices of genes based on homologous sequence analysis, it is necessary to specify the thresholds for amino acid sequence identity (set to 0.5 by default) and for the Smith–Waterman algorithm score used to filter homologous genes in the The thresholds to filter orthologous genes option

In the Additional parameters section, several additional analysis options can be selected: calculation of the divergence index (DI) in the DI analysis option, assessment of enrichment with single nucleotide polymorphisms (SNPs) and identification of Gene Ontology terms. For DI calculation, it is also possible to configure the groups of organisms for which the index is calculated in the dN /dS setup window. This option provides two configurations for the analysis. The first parameter, dN /dS level, defines the taxonomic level at which the dN /dS analysis is performed. This type of analysis is primarily used to compare sequences of closely related organisms. A value of 1 limits the analysis to organisms within a single genus. For example, when analysing human genes, a value of 2 indicates that the dN /dS will be calculated relative to other organisms in the Hominidae family. The second field, Organisms, allows you to enter specific species codes from the KEGG database. For example, to compare the sequence of a studied human gene not with all hominids but only with gorillas, the code “ggo” should be entered in this field

The output of Orthoweb for these analysis modes will be an archive file containing a tabular text file with the following data columns: Gene – KEGG gene identifiers, Label – Entrez gene identifiers, PAI – phylostratigraphic age index values; additional columns with values from supplementary analysis modes: DI, SNP and GO label.

**Analysis of gene group characteristics. **To calculate the Transcriptome Age Index (TAI) and the Transcriptome Divergence Index (TDI), it is necessary to select the input data format option Network file – Use expression. In this mode, the user must provide a tab-delimited text file containing one column of gene names and several columns of normalised expression values, labelled according to the experimental conditions under which the expression analysis was performed. The input file can be either a gene network file or simply a list of genes

As output, the Orthoweb program generates a tab-delimited text file with three columns: Data – with the names of the conditions specified in the input file, TAI – with the transcriptome age index values for the selected set of genes, and TDI – with the transcriptome divergence index values under the given conditions.

**Gene network analysis.** In addition to the analysis of indices for individual genes and gene lists, Orthoweb implements phylostratigraphic analysis and visualization of gene networks. Users can analyse networks imported from the KEGG Pathway (Kanehisa et al., 2017) and WikiPathways databases, as well as networks uploaded from text files. Access to network analysis from these databases is provided via the following link: https://orthoweb.sysbio.cytogen.ru/ pathway.html

Orthoweb supports import and analysis of networks from two major databases. The first supported database, KEGG Pathway, contains numerous gene networks and pathways classified according to various criteria such as metabolism, organismal system functions and human diseases. To start the analysis, the user must specify the pathway code and the organism for which the network is to be imported. As an output of network analysis from KEGG Pathway, Orthoweb will generate a gene network where the nodes display PAI values determined based on the KO groups present in the network. Since all elements in KEGG networks are described in the KEGG database itself, importing and analysing such networks is very convenient for Orthoweb, which retrieves most of the information needed for analysis directly from KEGG.

**Fig. 3.**
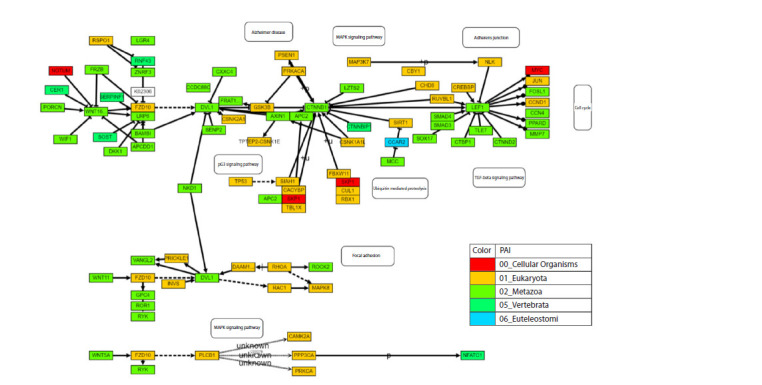
Example of network visualization from the KEGG Pathway database for the “Wnt signalling pathway” (https://www.kegg.jp/pathway/hsa04310), analysed using Orthoweb The color of each node corresponds to the PAI index of the gene (white elements represent pathways and chemical compounds). By default, the standard network structure is imported with preserved element coordinates, but the network scale can be adjusted by the user and each element can be interacted with.

As an example of this mode in Orthoweb, we analysed the Wnt/β-catenin signalling cascade network (Fig. 3). The Wnt/β-catenin signalling pathway is involved in the regulation of the cell cycle, adhesion, migration and differentiation. Activation of the pathway begins with the binding of WNT ligands to Frizzled and LRP receptors on the cell surface. This leads to the stabilisation and accumulation of β-catenin in the cytoplasm and its subsequent translocation to the nucleus, where it interacts with transcription factors and stimulates the expression of target genes (Davidson et al., 2009). Dysregulation of this pathway has been implicated in the development of several cancer types (Zhan et al., 2017). This signalling cascade is one of the most ancient signalling pathways and predominantly involves genes that originated during the emergence of multicellular organisms and eukaryotes (PAI = 1, 2).

The second database available for network import is WikiPathways. The networks presented in WikiPathways contain more details, entities and interaction variants compared to KEGG, which makes their complete import more difficult and requires the consideration of identifiers from several different databases.

**Fig. 4.**
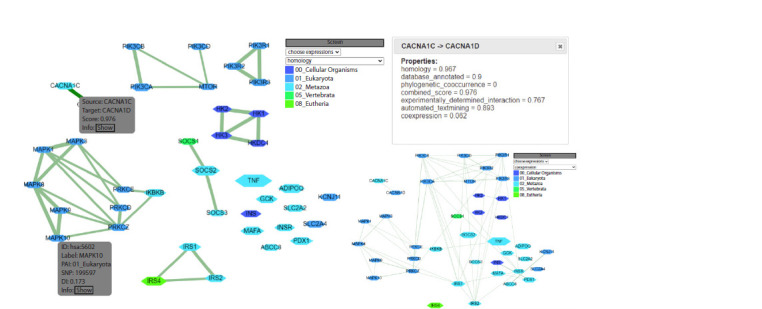
Example of a network imported from the STRING tool, where the color of each node corresponds to its PAI index and the thickness of the edges represents the combined_score value from STRING. By selecting a specific interaction within the network, information about the confidence levels of that interaction in STRING is provided

Orthoweb provides a step-by-step process for importing and analysing user-generated gene networks. Users can first import a network in TSV format (a tab-delimited text file) and then interact with it, e. g. rearrange elements, before starting the analysis. This format is compatible with the widely used STRING tool (von Mering et al., 2005), ensuring seamless integration of STRING data into Orthoweb without additional processing. For networks imported from STRING, the combined_score column contains the reliability of identified interactions, with weights ranging from 0 to 1. Upon completion of the analysis, the gene colours are updated to reflect their PAI values (Fig. 4). If additional analysis modes described earlier in the text are enabled, they will also be reflected in the visualization.

Database for storing results

To speed up index calculations and avoid redundant recalculations, Orthoweb includes a database containing tables for organisms, genes, pre-calculated PAI indices, DI indices, Gene Ontology terms (identifiers and names), SNPs and PAI indices determined based on KO groups. In addition to its use in interactive mode, this database can also be accessed via an API (Application Programming Interface) for integration with modelling environments or common scripting languages (Matlab, Octave, R, Python, etc.). This provides access to all available information on calculated PAI and DI indices for genes of specific organisms, allowing users to build data selection and visualization workflows. The API allows database queries to be made via specially structured URLs. Query results are returned as a structured text file in JSON format. A description of the API query keys and an example query to the database can be found in the Supplementary Material1.

## Conclusion

In this article, we present Orthoweb – a software platform designed for the analysis of phylostratigraphic and divergence indices for both individual genes and gene networks. Orthoweb also allows the integration of evolutionary index values with gene expression data under different conditions.

One of the key features of Orthoweb is its advanced data visualization capabilities. The tools for mapping evolutionary indices onto gene networks greatly simplify the interpretation of complex evolutionary relationships, making the results of analysis accessible to a wide range of researchers.

## Conflict of interest

The authors declare no conflict of interest.
